# Cannabis use among hospitalised adolescents before and after decriminalisation in South Africa

**DOI:** 10.4102/sajpsychiatry.v30i0.2244

**Published:** 2024-07-26

**Authors:** Michelle C. Swartz, Lisa Dannatt, Anusha Lachman

**Affiliations:** 1 Department of Psychiatry, Faculty of Health sciences, University of Cape Town, Cape Town, South Africa; 2 Department of Psychiatry, Faculty of Health sciences, Stellenbosch University, Cape Town, South Africa

**Keywords:** cannabis, adolescents, mental illness, high court ruling, decriminalise, South Africa

## Abstract

**Background:**

Cannabis is the most widely used substance worldwide, and its use is much higher among adolescents. However, adolescents are at higher risk of negative sequelae secondary to this use, including the possible development of mental disorders. On 31 March 2017, the South African High Court ruled that cannabis use by an adult in a private dwelling should be decriminalised.

**Aim:**

This study aimed to determine the clinical profile of adolescents who use cannabis, who use, who present to a tertiary hospital in Cape Town, South Africa, before and after the high court ruling in 2017.

**Setting:**

Folder review of adolescents admitted at Groote Schuur Hospital (GSH) in the Emergency Psychiatric Unit.

**Methods:**

This study was a retrospective folder review of adolescents admitted from April 2015 to March 2019.

**Results:**

Cannabis was the most commonly used substance in the study, with increased use seen post-ruling. The most common frequency of cannabis use reported was daily. A significantly higher proportion of patients who used cannabis pre-ruling had psychotic disorder (*p* < 0.001) and cannabis use disorder (*p* = 0.01). Post-ruling, the results were statistically significant (*p* < 0.001) for both.

**Conclusion:**

The study showed an increasing prevalence of cannabis use in adolescents admitted with mental illness after the high court ruling in 2017. This study also demonstrates that adolescents remain a vulnerable population to the effects of cannabis and highlights the need for further research.

**Contribution:**

The findings of this study call for more focussed adolescent interventions and services.

## Introduction

Cannabis is the most widely used drug globally, with an estimated 219 million users in 2021.^1,2^ Its use is particularly high among adolescents and youth.^3^ The cannabis use perception index showed that countries in Africa fell into the top two regions, following countries in Asia, which had the greatest increase in cannabis use.^1^ Cannabis has become more socially accepted and thought to be harmless, and with its changing legal status, it has allowed for more widespread exposure.^4,5^ Over the past few decades, cannabis has become more potent, with an increasing delta-9-tetrahydrocannabinol (THC) percentage.^6^ In the United States, the THC percentage increased from 4% to 16% between 1995 and 2018.^6^ Despite this, there has been a significant decline in the percentage of adolescents who consider regular cannabis use to be harmful.^6^ The risks of cannabis use are the highest in certain groups, including people with pre-existing mental disorders and adolescents.^5^

In South Africa, cannabis is the most commonly used illicit substance, with initiation reported as early as 11 years old.^4^ Factors that increase the risk of developing a substance use disorder (SUD) include socioeconomic inequalities, poverty, limited education and marginalisation, which are factors that many adolescents in South Africa are exposed to.^3^ Ongoing cannabis use during adolescence can result in long-term changes in brain function that can adversely impact educational, professional, and social achievements.^5^

Mental health conditions account for 16% of the global disease and injury burden in adolescents.^7^ In South Africa, violence, human immunodeficiency virus (HIV) infection, and substance use increase the risk of mental health disorders.^8^ The prevalence of cannabis use in adolescents with mental illness is high globally and in South Africa.^9^ To illustrate, a specific study conducted in Durban revealed that 61.4% of adolescents who were admitted because of psychotic symptoms reported having used cannabis at least once in their lifetime.^10^ Early initiation and frequent or heavy use of cannabis can lead to psychopathology.^11,12^ The exposure to cannabis during adolescence increases the risk for the development of psychosis in later life, and this risk is dose-related.^13,14^ Adolescent dual-diagnosis (psychiatric disorder with comorbid SUD) has multiple negative implications on social functioning, physical injury, sexual risk behaviour and the use of other illicit substances.^15,16,17^ A SUD negatively influences the course of mental illness and is often an indicator of poor treatment outcomes.^18,19^ A 2017 study in Durban found that over a third of adolescents with mental illness used substances, underscoring the urgent need for intervention in this vulnerable population.^19^

On 31 March 2017, the High Court of South Africa ruled that cannabis use by an adult in a private dwelling, where possession, purchase and cultivation are for personal consumption, should be decriminalised as it was inconsistent with the Constitution of South Africa.^20^ This ruling did not include adolescents in its judgement. According to Statistics South Africa, adolescence refers to the ages between 10 and 19.^21^ Changes in cannabis legislation can influence individual and population level initiation, frequency and quantity of use and lead to the progression of cannabis use disorder and its sequelae in vulnerable groups, which includes adolescents.^20,22^ Legislative changes should include policies that specifically protect adolescents to reduce the potential harms of cannabis use in this age group.^22^

This study reviewed cannabis use and the clinical profile of hospitalised adolescents before and after the legislative changes in South Africa. The data collected are crucial for understanding the needs of adolescents in the Groote Schuur Hospital’s (GSH) psychiatric services, identifying those with dual diagnoses, and enabling targeted substance intervention strategies.

## Hypothesis

It is hypothesised that, after the high court ruling in 2017, there would be an increase in cannabis use post-ruling, along with an increase in substance-related diagnoses.

## Aim

This study aimed to determine the clinical profile of adolescents who use cannabis and present to a tertiary hospital in Cape Town, South Africa, before and after the high court ruling in 2017.

## Research methods and design

### Study design

A retrospective, descriptive, cross-sectional folder review was used in this investigation.

### Study setting

The study was conducted by reviewing the folders of adolescents admitted at GSH in the Emergency Psychiatric Unit, Ward C23, in Cape Town, South Africa. The ward provides a specialised service for patients who require urgent psychiatric evaluation and management. The inpatient unit can accommodate up to 16 patients at a time, including adolescent, adult and geriatric patients. These patients are admitted under the *Mental Health Care Act* for a 72-h observation period. Patients who require further care after 72 h are referred to the relevant allied psychiatric hospitals.

### Study participants

The folders of all adolescents admitted from April 2015 to March 2019 were included in the study. These adolescents were admitted into a tertiary ward with a wide variety of mental disorders, with or without co-occurring substance use. To accommodate the unit’s admission criteria, the ages of adolescents included in the study were from 13 to 18 years. All adult and geriatric patients admitted into the tertiary ward from April 2015 to March 2019 were excluded from the study.

### Data collection

The following information was extracted from the folders and entered into a database:

- Socio-demographic details, including age, sex, and education.
- Substance history including cannabis, methamphetamines, alcohol and others.
- Psychiatric presentations, including admissions, discharge and referral.
- Psychiatric diagnosis (according to the *Diagnostic and Statistical Manual of Mental Disorders*, 5th edition, text revision 2022 [DSM-V-TR]^23^).
- Comorbid medical conditions.
- Stressors and social issues.
- Family history of substance abuse or psychiatric history.

### Data analysis

The statistical package SPSS version 27 was used to analyse the data. In order to describe the socio-demographic and clinical characteristics of adolescents using cannabis presenting to the unit, descriptive statistics were generated in the form of proportions (for categorical variables) and of means and standard deviations (for continuous variables). Comparative statistics were used as chi-square tests or Fisher’s exact tests where appropriate to compare outcome variables pre- and post-ruling. Psychiatric diagnoses and stressors were associated with cannabis use using chi-square tests. Because all data are categorical, normality does not apply.

### Ethical considerations

Ethical approval for this study was obtained from the Human Research Ethics Committee (HREC 398/2021) at the University of Cape Town, Faculty of Health Sciences. Groote Schuur Hospital granted permission to conduct this study.

The researcher did not interview patients and data collection was completed via folder review. Confidentiality was ensured by removing non-essential identifying information (names, initials, and hospital numbers), and the data were collected and stored using assigned research numbers. Data were saved on a password-protected excel workbook on a password-protected device.

## Results

The study period was divided into the pre-ruling period from April 2015 to March 2017 before the Western Cape High Court ruling that cannabis use for personal consumption should be decriminalised^20^ and the post-ruling period from April 2017 to March 2019.

The total number of adolescents admitted during the study period was 266. Of these, 40.5% (*n* = 108) of participants were admitted during the pre-ruling period and 59.4% (*n* = 158) were admitted during the post-ruling period. There was a significant increase in the number of females admitted post-ruling. Most of the admissions were first-time (*n* = 199; 74.8% overall), with 86.1% pre-ruling and 67.1% post-ruling.

Table 1 reports the demographic characteristics of the sample. The majority of admissions were between the ages of 15 and 16 pre- (*n* = 49; 45.3%) and post- (*n* = 67; 42.2%) ruling. Most adolescents (*n* = 182; 68%) were still attending school, with 59.2% of participants pre-ruling and 74.7% post-ruling. A small number of adolescents, 3% (*n* = 7), had completed school.

**TABLE 1:** Demographic data of the sample.

Demographic characteristics	Pre-ruling *N* = 108 (40.5%)				Post ruling *N* = 158 (59.4%)			
	*n*	%	Mean	s.d.	*n*	%	Mean	s.d.
**Sex**								
Male	73	67.6	-	-	67	42.4	-	-
Female	35	32.4	-	-	91	57.6	-	-
**Age (years)**	-	-	-	-	-	-	-	-
13–14	-	-	14	13	-	-	31	19.6
15–16	-	-	49	45.3	-	-	67	42.4
17–18	-	-	45	41.7	-	-	60	38.0
**Highest level of education**								
Still in school	64	59.2	-	-	118	74.7	-	-
Dropped out	28	26.0	-	-	35	22.1	-	-
Completed	2	1.8	-	-	5	3.2	-	-
Data unavailable	14	13.0	-	-	0	-	-	-
**Number of admissions**								
First presentation	93	86.1	-	-	106	67.1	-	-
Two or more	15	13.9	-	-	52	32.9	-	-

s.d., standard deviation.

Where family history data were available, 65% of patients (*n* = 126) had no history of psychiatric or substance use diagnoses. Seventeen per cent of patients (*n* = 32) had a family history of substance use problems, and 14% (*n* = 27) had a family history of psychiatric diagnosis. Five per cent of patients (*n* = 9) had a family history of both psychiatric and substance use diagnoses. It is also important to observe that family history data were unavailable for over a quarter of the patients.

Where medical history was available (*n* = 259), the majority (*n* = 216; 83%) had no comorbid conditions, and a minority of patients (*n* = 19; 7%) had a history of epilepsy/head injury. For 2.6% (*n* = 7) of patients, no data for medical history was available.

Table 2 presents the substance use of the participants pre- and post-ruling. The total number of patients using cannabis who were admitted during the study period was 116, of which 35% (*n* = 41) used pre-ruling, while 65% (*n* = 75) used post-ruling. Where data were available for the age of onset of cannabis use (*n* = 76), the majority were between 13 and 18 years of age (*n* = 54; 71%), with 57% pre-ruling (*n* = 16) and 79% (*n* = 38) post ruling (see Table 3). Overall, just under one-third were below the age of 12 (*n* = 22; 28.9%). When looking at school-going adolescents with concurrent cannabis use, 51.22% (*n* = 21) of participants were in school pre-ruling and 68% (*n* = 51) post-ruling.

**TABLE 2:** Substance use history.

Substances	Pre-ruling (*N* = 90)		Post-ruling (*N* = 152)	
	*n*	%	*n*	%
Substance use	52	57.8	91	59.9
Cannabis	41	45.6	75	49.3
Methamphetamine	20	22.2	17	11.2
Alcohol	18	20.0	45	29.6
Methaqualone	5	5.6	5	3.3
Nicotine	15	16.7	37	24.3
Other	1	1.1	5	3.3

**TABLE 3:** Specific cannabis use history.

Variable	Pre-ruling *N* = 41		Post-ruling *N* =75	
	*n*	%	*n*	%
**Age of onset, years**				
12 and below	12	29.3	10	13.3
13–18	16	39.0	38	50.7
Data unavailable	13	31.7	27	36.0
**Frequency**				
Daily	12	29.3	31	41.3
2–3 times per week	5	12.2	5	6.7
Weekends	5	12.2	9	12.0
Monthly	1	2.4	4	5.4
Once off	2	4.9	1	1.3
Data unavailable	16	39.0	25	33.3
**Sex**				
Male	35	85.4	46	61.3
Female	6	14.6	29	38.7

**FIGURE 1:**
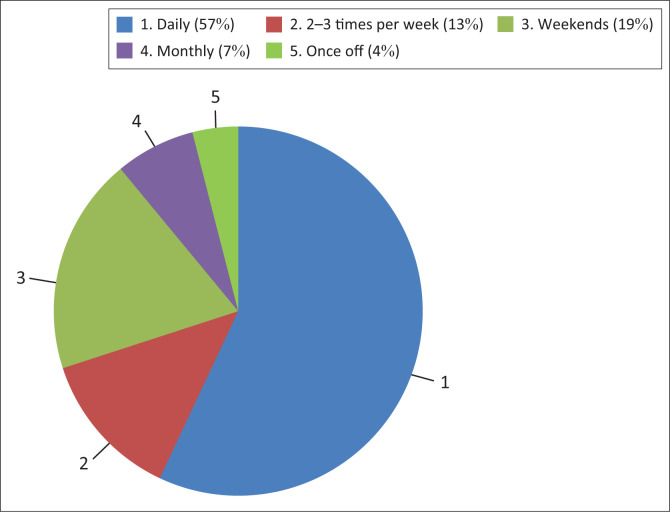
Frequency of cannabis use overall.

Data were only available for frequency of cannabis use in 75 patients (see Figure 1). Overall, the majority used cannabis daily (*n* = 43; 57%), with 48% (*n* = 12) pre-ruling and 62% (*n* = 31) post ruling (see Table 3). Documented urine toxicology results were only present for 24 patients.

### DSM-5-TR psychiatric diagnoses and stressors

The most frequent psychiatric diagnosis was trauma and stressor-related disorders (*n* = 130; 48.9%), followed by major depressive disorders (*n* = 105; 39.5%) and psychotic disorders (*n* = 88; 33.1%). Bipolar disorder was diagnosed in 6% (*n* = 16) and anxiety disorders in 3% (*n* = 7) of patients.

Fifty per cent (*n* = 113) of the sample had experienced family conflict and 27% (*n* = 61) experienced trauma. Twenty-two per cent of the sample (*n* = 50) reported no stressors. Other stressors included bullying, sexual identity, conflict with friends, and academic stress.

## Statistical analyses

### Pre-ruling

When comparing psychiatric diagnoses between those who used cannabis and those who did not, a significantly higher proportion of patients who used cannabis had a psychotic disorder (*p* < 0.001) and cannabis use disorder (*p* = 0.01). There were no other significant differences (see Table 4). Psychotic disorders (*n* = 27; 65.9%) were the most frequent psychiatric diagnosis in cannabis users, whereas in non-cannabis users, depressive disorders (*n* = 20; 40.8%) and trauma and stressor-related disorders (*n* = 21; 42.9%) were the most common diagnoses.

**TABLE 4a:** Psychiatric diagnoses in cannabis and non-cannabis users: pre- and post-ruling admissions.

Psychiatric diagnosis	Pre-ruling cannabis use				Test statistics	Post-ruling cannabis use				Test statistics
	Yes (*N* = 41)		No (*N* = 49)		*p*	Yes (*N* = 75)		Yes (*N* = 77)		*p*
	*n*	%	*n*	%		*n*	%	*n*	%	
Psychotic disorders	27	65.9	13	26.5	< 0.001**	36	48.0	9	11.7	< 0.001**
Bipolar disorders	0	-	3	6.1	0.248	6	8.0	7	9.1	0.810
Depressive disorders	9	22.0	20	40.8	0.056	24	32.0	40	51.9	0.013*
Anxiety disorders	0	-	2	4.1	0.498	1	1.3	2	2.6	1.00
Trauma and stressor-related disorders	13	31.7	21	42.9	0.277	31	41.3	29	37.7	0.643
Cannabis use disorders	8	19.5	1	2.0	0.010*	25	33.3	0	-	< 0.001**
Other	1	2.4	3	6.1	0.623	6	8.0	9	11.7	0.446

Note: Please observe that the total numbers of cannabis and non-cannabis users in the table may not match the overall total. This discrepancy is because of some patients having multiple psychiatric diagnoses and/or stressors.

^a^Fisher’s exact test performed.

* , *p* < 0.05;

** , *p* < 0.001.

**TABLE 4b:** Stressors in cannabis and non-cannabis users: pre- and post-ruling admissions.

Stressors	Pre-ruling cannabis use				Test statistics	Post-ruling cannabis use				Test statistics
	Yes (*N* = 34)		No (*N* = 40)		*p*	Yes (*N* = 73)		Yes (*N* = 76)		*p*
	*n*	%	*n*	%		*n*	%	*n*	%	
Trauma	6	17.6	10	25.0	0.444	29	39.7	16	21.1	0.013*
Family conflict	20	51.3	19	48.7	0.331	51	69.9	23	30.3	< 0.001**
Bereavement	4	11.8	3	7.5	0.696	13	17.8	9	11.8	0.305
Single parent	11	32.4	9	22.5	0.342	16	21.9	19	25.0	0.657
Other	7	20.6	5	12.5	0.347	18	24.7	12	15.8	0.177
None	8	23.5	9	22.5	0.916	9	12.3	24	31.6	0.005*

Note: Please observe that the total numbers of cannabis and non-cannabis users in the table may not match the overall total. This discrepancy is because of some patients having multiple psychiatric diagnoses and/or stressors.

^a^Fisher’s exact test performed.

* , *p* < 0.05;

** , *p* < 0.001.

There were no significant differences between those who used cannabis and those who did not use it in terms of stressors experienced (see Table 4). In both cannabis users and non-users, the most frequent stressor experienced was family conflict (51.3% and 48.7%) and the least common stressor experienced was bereavement (11.8% and 7.5%).

### Post-ruling

When comparing psychiatric diagnoses between those who used cannabis and those who did not, a significantly higher proportion of patients who used cannabis had a psychotic disorder and cannabis use disorder (*p* < 0.001). A significantly higher proportion of patients who did not use cannabis had depressive disorder (*p* = 0.013). Psychotic disorders (*n* = 36; 48%) and trauma and stressor-related disorders (*n* = 31; 41.3%) were the most frequent psychiatric diagnoses in cannabis users. In contrast, in non-cannabis users, depressive disorders (*n* = 40; 51.9%) and trauma and stressor-related disorders (*n* = 29; 37.7%) were the most common diagnoses.

A significantly higher proportion of patients who used cannabis post-ruling reported experiencing trauma (*p* = 0.013) and family conflict (*p* ≤ 0.001). A significantly higher proportion of patients who did not use cannabis reported experiencing no stressors (*p* = 0.005). In both cannabis users and non-users, the most frequent stressor experienced was family conflict (69.9% and 30.3%), and the least common stressor experienced was bereavement (17.8% and 11.8%) (see Table 4).

## Discussion

This study examined the clinical profile of adolescents admitted to a tertiary psychiatric unit in Western Cape before and after the 2017 high court ruling to better understand the prevalence of use of cannabis, sequela of use, factors influencing use and protective factors. The study was completed in a single tertiary-level hospital in the Western Cape and included 266 participants. This study reported a high proportion of school-going adolescents, with 59.2% of participants in school pre-ruling and 74.7% post-ruling. Of those school-going adolescents with concurrent cannabis use, 51.22% of participants were in school pre-ruling and 68% post-ruling. This differed from findings in a non-clinical study of Western Cape schools in 2011, which showed 23.6% of adolescents reporting cannabis use.^24^ This could be attributed to the fact that cannabis-related adolescent hospitalisations are increasing with the changes in the legal status of cannabis.^25^

Most of the admissions were index presentations and there was an increase in the number of adolescents admitted into the unit post-ruling. The possible reasons for ongoing increases in admission rates may include societal and policy changes in a growing population.^26^ The society surrounding adolescents has changed from previous years in a way that places more demands on them, making coping with those demands harder than before and eventually leading to seeking psychiatric treatment or being referred.^27,28^ In the South African context, societal stressors such as high rates of crime, violence, poverty and substance abuse are factors resulting in psychiatric morbidity in children and adolescents.^29^ It is thought that a redefining of normal adolescent development has taken place, which was viewed as just behavioural problems but is now being recognised as psychiatric disorders. Another reason for an increased need for psychiatric inpatient care may be inadequately resourced outpatient and preventive services in adolescents.^26^

The most commonly reported substances used in the study pre-ruling were cannabis, methamphetamines, and alcohol. However, post-ruling, it was cannabis, alcohol and nicotine. These data differ from a 2011 non-clinical study in Western Cape schools, which showed that cannabis was the third most reported substance after tobacco and alcohol.^24^ Factors that can account for this difference in findings are that their sample was in a non-clinical setting. The population in our sample represents adolescents with psychiatric symptoms that required admission, and thus, the pattern of substance use may be different in this population. This is in keeping with a review of illicit drug use in South Africa, where the estimated cannabis use percentage in otherwise healthy adolescents was much lower than the rates of cannabis use found in the mentally ill population.^9,30^ More recent data from the South African Community Epidemiology Network on Drug Use (SACENDU) update of 2022, which looks at treatment data, showed that across the majority of sites in South Africa, except KwaZulu-Natal, most people under the age of 20 years (59.8% – 71.9%) reported that cannabis was their primary drug used.^31^ Another contributor could be increased cannabis use over time in the adolescent population.^32^ This was seen in a Canadian study looking at trends in youth cannabis use across cannabis legalisation.^32^ A cross-sectional analysis showed that cannabis ever-use was significantly higher in the year after legalisation compared to the year in which it took place, indicating an increase over time.^32^ The change in substance use trends that was seen in pre- versus post-ruling is also in keeping with international trends where, with the exception of nicotine and alcohol, cannabis is the most commonly used drug in young people.^1^ The reasons for this include the perceived ease of availability, together with the perceptions of low risk of harm in comparison to other substances.^1^

In our study, both pre- and post-ruling, cannabis was the most reported substance used. Our study also showed a 3.7% increase in adolescents who smoked cannabis when comparing data pre- and post-ruling, which indicates an increased prevalence not only of cannabis use but also of cannabis use disorders. This is in keeping with the World Drug Report of 2023, which states that the overall estimated number of annual cannabis users has increased by 21% over the past decade.^2^ In addition, there has been a notable increase in hospitalisation because of cannabis use disorders.^33^ The increase can be explained by the attitudes about cannabis use continuing to move towards greater acceptance.^34^ This could also be because of factors such as higher potency of cannabis products, increased frequency of use and changes in legislation.^33^ In our study, urine toxicology results were documented in a minority of patients. Drug testing in adolescents is important, as they may not be forthcoming, and it may be helpful when the history is negative in the context of clinical signs and symptoms suggesting substance use.^35^

There was a gender difference in our study pre- and post-ruling, with 67.6% of participants being male pre-ruling and 57.7% being female post-ruling. In the participants who used cannabis, the difference between males and females pre-ruling was 70.8%, whereas post-ruling was 22.6%. Thus, the proportion of females using cannabis increased post-ruling. According to the World Drug Report, the gender divide in cannabis use is reducing in some regions.^2^ Gender roles, which are defined by society and culture, can significantly influence the initiation and progression of substance use and the development of SUDs.^33^ The difference can also be attributed to differing opportunities for cannabis use in various environments rather than any inherent biological or psychological distinctions between men and women that might affect cannabis use and the progression of cannabis use disorders.^33^ Further research is required to explore the relationship between gender and cannabis use, which is complex and ever-evolving.^36,37^ Despite this difference, both pre-and post-ruling, the majority of the adolescents who used cannabis were male. In all three South African Youth Risk Behaviour Surveys, cannabis use was higher in males than females.^38,39,40^ Of note, in the small qualitative study referred to in the previous paragraph, all the participants included were male between the ages of 14 and 19 years.^41^ A possible reason why more males are found to use cannabis may be adherence to dominant male norms or male typicality, which was found to be associated with higher rates of substance use and dependence.^37^

Regarding DSM-5-TR diagnoses, findings both pre- and post-ruling showed that major depressive disorders and trauma and stressor-related disorders were the most common diagnoses in non-cannabis users. This is in keeping with the World Health Organization’s findings, where depression was found to be the most common cause of illness and disability in adolescents, and self-harm was listed among the top 10 causes.^42^ Depression and post-traumatic stress were found to be common in adolescents from poorer socio-economic conditions.^43^ The risk of mental illness is increased in environments with poor social support, particularly in low and middle-income countries (LMICs) with socio-economic inequalities, such as South Africa. In the South African context, factors that further increase vulnerability to mental health disorders include exposure to violence, HIV, infection and substance use.^8,43^

Trauma was reported as a stressor in cannabis users both pre- and post-ruling. However, a significantly higher proportion of patients who used cannabis post-ruling reported experiencing trauma, which included physical, verbal, sexual and neglect. This aligns with an association found in a Cape Town study of school-going adolescents, where childhood abuse was linked to increased alcohol/drug problems and drug use coping.^44^ In addition to a higher proportion of patients experiencing trauma as a stressor post-ruling, our study also observed a difference in psychiatric diagnoses. Psychotic disorders were found to be the most common diagnosis pre-ruling, but post-ruling, psychosis and trauma and stressor-related disorders were most common. A possible explanation for this could be the increased number of participants using cannabis post-ruling, which resulted in different findings. An American study found that higher rates of childhood trauma, lifetime trauma, and significant life events were found in cannabis users when compared to non-users.^45^ A possible explanation for the high rates of cannabis use among trauma-exposed individuals could be the dysregulation of the endocannabinoid system.^45^ Another possible explanation could be the increased proportion of females using cannabis post-ruling. According to the World Drug Report of 2018, women who experienced childhood adversity, such as physical neglect, abuse or sexual abuse, may use drugs to self-medicate. Furthermore, women with SUDs are reported to have high rates of post-traumatic stress disorder.^1^

There is mounting evidence that suggests a correlation between adolescents who regularly use cannabis and more severe and adverse outcomes compared to those who start using it during adulthood.^5,46^ In our study, both pre- and post-ruling, a significantly higher proportion of patients who used cannabis were diagnosed with a psychotic disorder and cannabis use disorder. Studies of psychosis have indicated that specific individuals are more susceptible to the effects of cannabis than others. These include those who started using cannabis at an early age, those who use it frequently, and those who use a more potent form of cannabis.^9^ Where data were available in our study for the frequency of cannabis use, daily use was reported in more than half of the participants. Daily use of cannabis, especially of high potency, contributes to an earlier onset of psychosis in cannabis users.^47^

## Limitations

There were several limitations to this study:

- This was a retrospective folder review, which was reliant on clinical notes and may have been influenced by the interviewer’s experience and training.
- Not all patients had a urine toxicology test documented. These were requested in most adolescents admitted, but tests were only sometimes available because of budget constraints. This is an important factor as, in most cases, we were unable to confirm the substance history with a test. The timing of testing is also important. If these tests are not performed on the day of admission, they may result in a false negative when conducted later. This can influence the reliability and validity of the study.
- This study was conducted in a single hospital in the Western Cape with a relatively small cohort of patients; therefore, generalisability to the greater adolescent population may be limited.

## Recommendations

The authors suggest the following recommendations that could help improve the management of adolescents with comorbid mental illnesses and cannabis use presenting for hospital care. The recommendations include the need for dual diagnosis programmes for adolescent patients with mental health and cannabis use disorders. This would assist in improving the prognosis and reduce the rates of re-hospitalisation. In resource-constrained settings, like South Africa, this service would be difficult to establish and requires strategic planning and budget allocation from the Department of Health. With cannabis use increasing among youth, strategies and interventions for reducing and preventing cannabis use are essential in this group to reduce the health risks and disease burden. This would include health promotion and education programmes targeted at all adolescents.

## Conclusion

This study demonstrates that adolescents remain a vulnerable population to the effects of cannabis, with the increasing prevalence of cannabis use in adolescents admitted with mental illness. This study was limited by a single hospital. However, it highlighted the need for ongoing research on adolescents regarding their mental health burden and substance use, especially cannabis, within the South African context considering the changing legislation. In addition, this emphasises the need for more focused adolescent interventions and services. What is needed are more programmes on substance use prevention to increase awareness of the potential harms associated with cannabis use. Furthermore, screening of adolescents for SUD to provide early interventions to improve outcomes and the need for legislation aimed at protecting vulnerable individuals such as adolescents should also be considered.
